# Estimating the effect of international crude oil prices on RMB exchange rate with two-way fluctuation spillover

**DOI:** 10.1371/journal.pone.0292615

**Published:** 2023-10-10

**Authors:** Yuan Li, Jin Chen

**Affiliations:** School of Banking and Finance, University of International Business and Economics, Beijing, China; The University of Hong Kong, HONG KONG

## Abstract

International crude oil prices has become one of the important factors that affect exchange rate fluctuation. By combining TVP-VAR model and DY time-varying spillover index, this paper measures the two-way fluctuation spillover effect between international crude oil prices and RMB exchange rate from 2011 to 2022. The research results show that: (1) There are obvious time-varying characteristics among international crude oil prices, onshore RMB exchange rate and offshore RMB exchange rate, and they are strengthened when major events occur at home and abroad; (2) The spillover and absorption level among international crude oil prices, onshore RMB exchange rate and offshore RMB exchange rate is relatively limited; (3) After the “exchange rate reform on August 11”, the market participation of RMB exchange rate has increased year by year, and the offshore RMB is mainly net spillover, while the onshore RMB is mainly reflected as a recipient; (4) The offshore RMB exchange rate has a driving effect on the onshore RMB exchange rate. Based on this, it is necessary to orderly promote the development and utilization of new energy, actively promote the market-oriented reform of RMB exchange rate, play the leading role of offshore market, and accelerate the construction of crude oil-RMB settlement system.

## I. Introduction

As the first crude oil importer, the sharp fluctuation of international crude oil prices, the excessive dependence of China’s economy on crude oil and the basic resource role of crude oil have become important factors affecting macroeconomic stability of China [1]. According to the public data of the National Bureau of Statistics, since 2019, the net import scale of crude oil of China has remained at about 500 million tons and exceeded 510 million tons in 2021, an increase of about 90% compared with 2012. In addition to the obvious increase in the total scale, China’s energy structure also has the characteristics of “rich in coal resource, poor in oil resource, less in gas resource”. In 2021, China Petrochemical News stated that China’s crude oil import dependency reached a high level of 72%, which means that the current gap between crude oil demand and supply is still large. In this context, the fluctuations in international crude oil prices, especially rapid increases in the short term, will inevitably transmit to the oil-related industries and sectors. This results in increased costs for crude oil imports, hindered exports, and companies facing profit reduction expectations and weak consumer demand. Ultimately, this puts pressure on the RMB exchange rate, negatively impacting economic development and financial stability. Considering China’s current significance in global trade, crude oil, and the economy, the effects of decreased export capacity, diminished import demand, and declining crude oil demand will be transmitted to other countries, particularly those in China’s upstream and downstream industries.

In light of this, the article employs a TVP-VAR-DY model to construct a time-varying spillover index that incorporates international crude oil prices and the RMB exchange rate. This model not only adequately accounts for extreme volatility but also offers the advantage of objectively and flexibly measuring the nonlinear relationship between the two variables. This is in line with the research objectives of this paper.

## II. Review of relevant research literatures

Both crude oil prices and exchange rate are the important basis and guarantee of the macroeconomic stability of a country. The existing research has rich and detailed materials on the mutual influence and interaction between them. By sorting out the content related to the research objective of this paper, it can be divided into the following three categories.

From the results of foreign studies, it’s widely believed that there is an obvious linkage relationship between crude oil prices and exchange rate, but there is no consensus on the research conclusions. Krugman [2] was a scholar who paid earlier attention to the relationship between crude oil and the US dollar exchange rate by building a partial equilibrium portfolio model including the US, Germany and the Organization of Petroleum Exporting Countries (OPEC). They point out that in the short term, higher oil price will promote dollar appreciation, but in the medium to long term, the dollar will devaluate. Amano and Van Norden [3] analyzed the relationship between international crude oil prices and the US dollar from the perspective of terms of trade, and they believed that crude oil prices increasing 1% would lead to the US dollar increasing 0.513%. Contrary to Amano and Van Norden [3], Lizardo and Mollick [4] concluded that international crude oil prices had a significant negative effect on the US dollar. In addition to the US dollar, Antonio and Luis [5] also focused on the euro area, and after making analysis by the establishment of Phillips curve including crude oil prices, they concluded that the rise of crude oil prices had positive effect on the euro. At the same time, the appreciation of the euro had indirectly suppressed the impact of higher crude oil prices on eurozone inflation by about 15%. In addition to paying attention to the impact of crude oil prices on exchange rate, some scholars also began to pay attention to the impact of exchange rate changes on crude oil prices, such as Brahmasrene et al. [6], Albulescu and Ajmi [7] and Jung et al. [8]. Brahmasrene et al. [6] focused on the relationship between the US crude oil import price and the bilateral exchange rates of the five major export countries—Canada, Mexico, Colombia, the United Kingdom and Venezuela—from 1996 to 2009. They believed that the exchange rate had a significant negative effect on the price of crude oil. Albulescu and Ajmi [7] conducted a causal test by using a recursive method and found that there was a mutual causal relationship between crude oil prices and exchange rate, which was further strengthened by the outbreak of the international financial crisis in 2008. By using the NARDL model, Jung et al. [8] found that when the Canadian dollar depreciated by 1% and appreciated by 1%, the price of crude oil correspondingly rose by 2.22% and fell by 1.79%.

With the acceleration of China’s opening up and the continuous improvement of the market-oriented level of exchange rate, domestic scholars have also begun to pay attention to the fluctuation of RMB exchange rate under the fluctuation of crude oil prices. The most research results show that there is a negative relationship between crude oil prices and RMB, such as Zhang Qingjun [9], Ding Xuhui et al. [10], Tan Dekai and Tian Lihui [11]. Zhang Qingjun [9] analyzed the dynamic relationship between the real effective exchange rate of RMB and international crude oil prices from 2001 to 2010. They believe that in a short period of time after the change of crude oil prices, the real effective exchange rate of RMB will have a significant negative result. Ding Xuhui et al. [10] pointed out that when the international crude oil prices increased by 1%, RMB exchange rate would correspondingly decrease by 0.11%. However, Tan Dekai and Tian Lihui [11] believe that the existence of this negative relationship is conducive to establishing a RMB pricing mechanism in crude oil trade. Li Jianfeng et al. [12] used VAR-BEKK-MGARCH model to verify the relationship between crude oil and exchange rate, and the results showed that there was a two-way spillover relationship between international crude oil prices and RMB exchange rate. In consideration of the impact of crude oil prices on RMB exchange rate, some scholars began to pay attention to the transmission mechanism and its role between the two, such as Zhou Donghai et al. [13]. Zhou Donghai et al. [13] established a TVP-SV-VAR model with four variables including crude oil prices, exchange rate, interest rate and Chinese stock market under the Bayesian framework. The research results show that the impact of crude oil prices on exchange rate is very significant, and stock price and interest rate play different roles in the transmission process, and the transmission efficiency of stock price is significantly higher than that of interest rate. They believe that this is because China’s interest rate liberalization is still in the process of reform, and its price transmission mechanism has not fully played its role.

Due to the presence of time-varying issues and highly volatile research subjects, the research in this area primarily focuses on securities markets, exchange rate markets, and interest-rate policies. Many scholars have used GARCH models and VAR models to construct empirical testing models. For instance, Jia et al. [14] employed the DCC-GARCH model to examine the time-varying characteristics of contagion in the stock markets of Mainland China, Japan, Hong Kong, and Singapore. They found evidence of financial contagion among Asian stock markets, with this relationship being more significant during international financial crises. Wu et al. [15] also studied the time-varying relationships in stock markets, using the LASSO-VAR model to construct a model based on the return rates of 36 market indices from 2006 to 2015, confirming the existence of financial market co-movement phenomena. Chen et al. [16] focused on the Shanghai Stock Exchange A shares and explored causal relationships under both linear and nonlinear Granger conditions using a dynamic network graph as a framework. Sui et al. [17] analyzed and examined the exchange rate relationships among countries along the Belt and Road Initiative, utilizing the DCC-GARCH model to characterize the dynamic influence of the Chinese yuan. Shang et al. [18] focused on the high-frequency time-varying characteristics of price-based and quantity-based monetary policies, employing the MF-TVP-FAVAR model in their analysis of influencing factors.

After reviewing the existing research, the research experience of domestic and foreign scholars on the relationship between crude oil prices and exchange rate fluctuation provides strong theoretical support for this paper. However, the above researches still have room for further improvement: Firstly, the research method is a linkage effect analysis mainly based on the GARCH model and VAR model. Although the interrelationships among the variables have been effectively measured, however, above model has shortcomings, such as difficulty in accurately grasping non-linear relationships, requirements for sample data volume, and difficulty in understanding the interaction of real variables, which cannot meet the research needs and objectives of this paper. Secondly, most current studies focus on the RMB/US dollar exchange rate or real effective exchange rate, and few studies take the special situation of “one market, two exchange rates” in China into account. Thirdly, few studies focus on the different impact of between onshore RMB exchange rate and offshore RMB exchange rate on crude oil prices. Therefore, the following two aspects of the tentative research were carried out in this paper: (1) expanded the research methods. Through the TVP-VAR-DY model, the application of this model can effectively overcome the deficiencies of the above model. Not only can the model autonomously select the window to ensure the objectivity of the analysis results, but the full use of the whole sample data can effectively capture the changes under different economic and financial conditions. More importantly, it can realize the different changes of the overall and individual variables, establishing a comprehensive relationship framework; (2) Increased the dimension of the research content. RMB exchange rate was decomposed into onshore RMB exchange rate and offshore RMB exchange rate, which improved the accuracy of the research results.

## III. The theoretical mechanism of the influence of international crude oil prices on RMB exchange rate

From the perspective of theoretical mechanism, the transmission process of international crude oil prices to RMB exchange rate is complicated and long-term. As the most important industrial raw material, crude oil is closely related to economic life. When the price of crude oil changes, it will play a role in the RMB exchange rate through different mechanisms [1, 19, 20].

### 1. Transmission mechanism of international crude oil prices to RMB exchange rate

**Fig 1:**
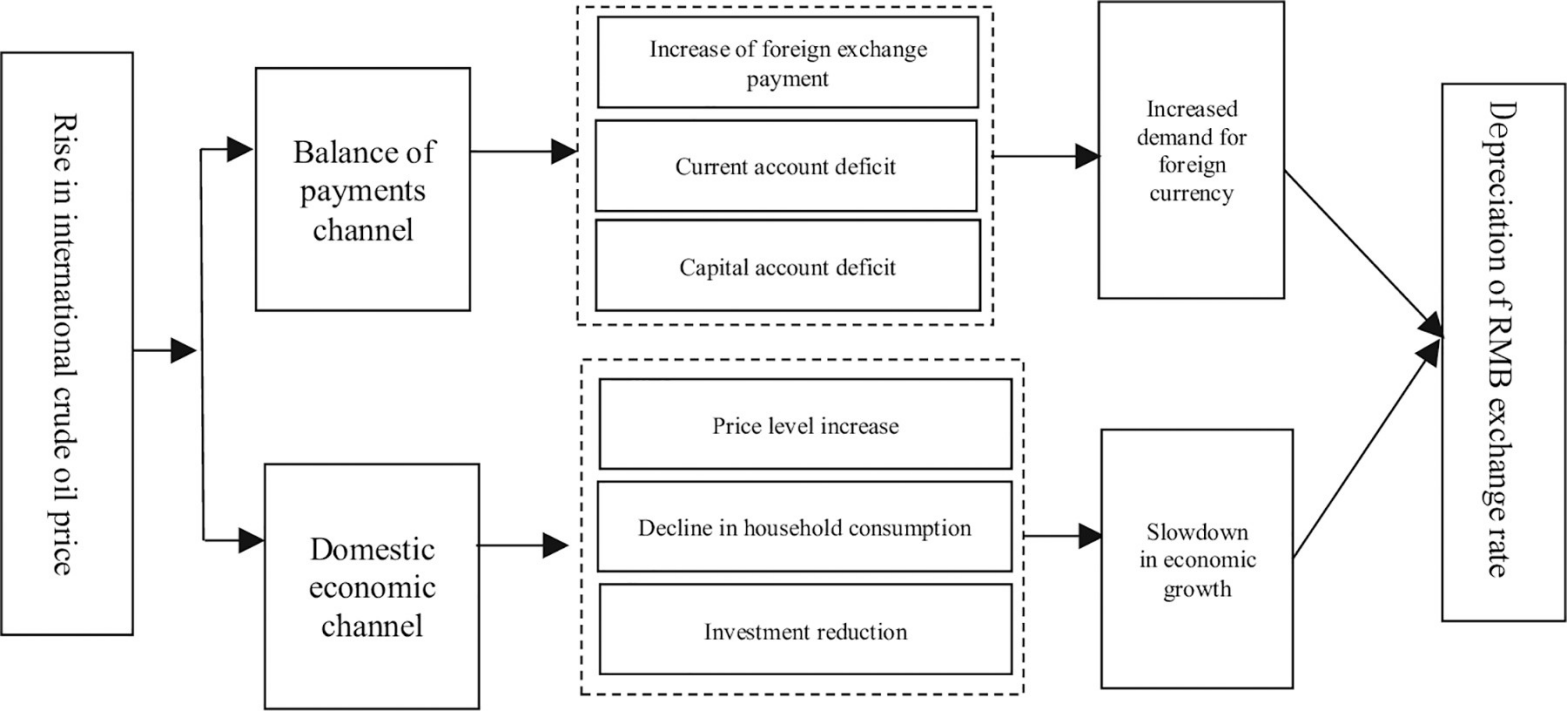
Transmission mechanism of international crude oil prices to RMB exchange rate.

This paper takes the rise of international crude oil prices as an example. The change of international oil price mainly affects RMB exchange rate market through two paths of international balance of payments and domestic economy, and the relationship between the two is negative in general. The details are shown in Fig 1.

#### 1. Channels for international balance of payments

Due to the existence of the oil-dollar system, when the price of crude oil denominated in dollars rises, China needs to pay more dollars to complete the transaction settlement, and the demand for dollars increases rapidly in short term, causing additional foreign exchange payment. Other things being equal, increased demand for dollars will result in the relative depreciation of RMB. The rise of crude oil prices will also bring pressure on the import cost of crude oil for relevant industries and sectors. The rise of product cost will make sales price rise simultaneously, which will not help Chinese enterprises to maintain their price advantages and competitiveness in international trade. The decline of export may result in trade deficit and reduce the real demand for RMB in the international market. Of course, the degree and size of changes in import demand mainly depend on the elasticity of crude oil import demand. It is worth noting that once the rise of crude oil prices forms market expectation, this expectation will intensify the preference of domestic market for foreign currencies, especially for US dollar, which will trigger short-term capital outflows, resulting in pressure on RMB.

#### 2. Domestic economic channels

Change of international crude oil prices will have an impact on important domestic economic indicators, and change of macroeconomic fundamentals will also have an impact on RMB exchange rate, and such impact will last further. When the international crude oil prices rise, it will have an adverse impact on related industries and alternative industries based on crude oil, and the increase of crude oil prices will be passed from upstream industries to downstream industries, resulting in the rise of producer price index (PPI) and consumer price index (CPI), and imported inflation will reduce the real purchasing power of RMB. Furthermore, the rise of crude oil prices will prompt relevant domestic enterprises to seek resources that can replace crude oil, such as coal, hydropower and natural gas, etc., which will increase the market price of energy and cause the overall price level of the society to increase. In this case, the consumption capacity and willingness of domestic residents will decline under the influence of prices, and the reduction of total social demand will not be conducive to domestic economic development. The superposition of three factors, including decline of export scale, decline of consumer expenditure and high price level, will inevitably result in the decline of the willingness of domestic enterprises to make new investment and reinvestment, and RMB depreciation will be expected to increase.

### 2. Transmission mechanism of RMB exchange rate to international crude oil prices

In the 1970s, the impact of the war on international crude oil prices was increasing sharply, the United States took the opportunity to sign an oil-dollar agreement with the Saudi government to determine use the US dollar as the main means of payment. In recent years, with the rise of China’s economy and the promotion of the international status of RMB, the steady appreciation of RMB can improve the purchasing power of China in the international crude oil market. However, as the import scale is relatively limited compared with the world import scale and the US dollar is still the main currency of international commodity pricing transactions, it is difficult for RMB exchange rate fluctuations to have a significant impact on international crude oil prices in the short term.

From the above analysis on transmission path, international crude oil prices change mainly have an impact on RMB exchange rate currently, and the transmission mechanism includes foreign exchange expenditure, current account, capital account, price level and consumption level, etc. The rise of crude oil prices has a negative effect on the RMB.

## IV. Construction of the model on the impact of international crude oil price on RMB exchange rate

### 1. Model construction

By virtue of the research idea of Antonakakis et al. [21], this paper measures the two-way fluctuation spillover effect between international crude oil price, onshore RMB exchange rate and offshore RMB exchange rate. Antonakakis et al. [21] transformed the TVP-VAR model into the TVP-VAR-DY model on the basis of Diebold and Yilmaz [22]. The constructed TVP-VAR-DY model achieves the following three advantages: (1) it ensures the integrity of all data and provides more accurate analysis results; (2) it fully takes the impact of outliers on variable fluctuation into account, especially the short-term impact of extreme events; (3) Because there is no need to set the time window of rolling window, the human subjectivity is effectively reduced. In this model, the total spillover index, directional spillover index and pairwise net spillover index are provided. The total spillover index formula is shown in (1):

$$Q_{t}(H)=\frac{\sum_{i,j=1,i\neq j}^{m}{\tilde{\phi }}_{ij,t}(H)}{\sum_{i,j=1}^{m}{\tilde{\phi }}_{ij,t}(H)}*100$$ (1)

Where, ${\tilde{\phi }}_{ij,t}(H)$ represents the contribution that over spills from variable *j* to variable *i*. In addition to the total spillover index, Eqs (2) and (3) represent the influence of variable *i* on variable *j* and the influence of variable *j* on variable *i*, respectively

$$Q_{i\to j,t}(H)=\frac{\sum_{j=1,i\neq j}^{m}{\tilde{\phi }}_{ji,t}(H)}{\sum_{j=1}^{m}{\tilde{\phi }}_{ji,t}(H)}*100$$ (2)

$$Q_{i\leftarrow j,t}(H)=\frac{\sum_{j=1,i\neq j}^{m}{\tilde{\phi }}_{ij,t}(H)}{\sum_{i=1}^{m}{\tilde{\phi }}_{ij,t}(H)}*100$$ (3)

In order to measure the self-spillover level of variables in the market, Antonakakis et al. [21] obtained the net spillover index of variable in the whole system by establishing the difference between equation (2) and equation (3). The calculation formula is as follows:

$$Q_{i,t}=Q_{i\to j,t}(H)-Q_{i\leftarrow j,t}(H)$$ (4)

If the result in equation (4) is positive, it means that the spillover level of variable *i* to other variables is greater than the spillover level of other variables to itself, which indicates that this variable has a significant impact on the market. In contrast, if variable *i* is negative, which indicates that the variable is the recipient of spillover effect of other variables in the market.

### 2. Data selection and descriptive statistical analysis

This paper selects the daily data of international crude oil prices, onshore Renminbi exchange rate, and offshore Renminbi exchange rate from January 4, 2011 to September 30, 2022 as the research objects, and the corresponding data are the spot price of Brent crude oil, the central parity rate of RMB against the US dollar and the non-deliverable forward exchange rate of RMB. All data with intersection in retention time totally get 2675 observed values. After the outbreak of the international financial crisis in 2008, the People’s Bank of China returned to the Renminbi-US dollar peg system to prevent excessive fluctuations during the crisis, which could not reflect the actual economic situation. In June 2010, the marketization reform of the Renminbi exchange rate was put back on the agenda. Considering the lagging effect of policy implementation, this paper sets the starting point of the study in 2011.All data is from the WIND database. Before using the TVP-VAR-DY model for estimation, it is necessary to make sure that all data can meet the stationarity requirements. Therefore, this paper draws on the existing research experience and processes the data according to the formula to get the volatility.

From Table 1, the skewness result indicates that the crude oil price in the selected data presents left-skewed distribution, while the two RMB exchange rates present right-skewed distribution. The kurtosis of the three variables is all greater than the value 3 of the normal distribution, and the offshore RMB exchange rate reaches the maximum value of 60.461, which means that the data series does not meet the normal distribution. Jb-test results again show that the data is not normally distributed, but meets the “sharp peak and heavy tail” pattern. All the data in ADF stationarity test reject the null hypothesis requirement and can be followed up.

**Table 1:** Descriptive statistics of variables and unit root test.

Variable name	Variable symbol	Mean value	Standard deviation	Skewness	Kurtosis	JB-test	ADF test
**International crude oil prices**	BRENT	0.033	2.652	-0.172	22.890	44108.51***	-10.633***
**Onshore RMB exchange rate**	CNY	0.003	0.202	0.742	13.037	11473.45***	-8.464***
**Offshore RMB exchange rate**	NDF	0.004	0.333	0.729	60.461	368248.60***	-9.092***

Note: ①***, **, *indicate the significance level at 1%, 5% and 10% respectively; ② ADF unit root test is the test result with trend term and constant term; ③When the number of decimal places is higher than three, only 3 decimal places are reserved.

## V. Empirical analysis of the impact of international crude oil price on RMB exchange rate

### 1. Static spillover effect analysis

Based on the SBIC principle, this paper determines that the optimal lag order of this model is 2, and the decomposed number of stages in predicting error variance is consistent with the selection of Guo Na and Zhang Jun [23]. Static spillover effect results were obtained by setting parameters nlag = 2 and nfore = 10 in the TVP-VAR-DY model (see Table 2).

**Table 2:** Static spillover effect of international crude oil prices, onshore RMB exchange rate and offshore RMB Exchange rate (%).

	BRENT	CNY	NDF	FROM	TCI
**BRENT**	96.2	1.3	2.5	3.8	
**CNY**	2.8	68.1	29.0	31.9	
**NDF**	2.8	16.4	80.8	19.2	
**TO**	5.6	17.7	31.6	54.9	
**NET**	1.8	-14.2	12.4		
**TCI**					18.3

The average value of spillover index of international crude oil prices, onshore RMB exchange rate and offshore RMB exchange rate are reported in Table 2 respectively. In addition to the results of total spillover index (TCI), directional spillover index (From and To) and net spillover index (Net), the diagonal line of the table shows the impact of variables on themselves. According to the results in Table 2, the following research conclusions can be obtained: Firstly, the average value of the total spillover index composed of the three variables is 18.3%, indicating that the linkage between the current international crude oil price and the RMB exchange rate is stable and tight to some extent. Secondly, in this model, the impact of international crude oil price, onshore RMB exchange rate and offshore RMB exchange rate on themselves is 96.2%, 68.1% and 80.8% respectively, which means that the impact of international crude oil prices on themselves is relatively significant, while the fluctuation of RMB exchange rate can be affected by 20%-30% external factors. Thirdly, from the spillover level (To) and net spillover level (Net) results of RMB exchange rate, the spillover level of offshore RMB exchange rate is significantly higher than that of onshore RMB exchange rate. In contrast to the absorption level (From), the offshore RMB exchange rate is influenced by external factors at 19.2%, which is smaller than the onshore RMB exchange rate at 31.9%.

### 2. Dynamic total spillover effect analysis

**Fig 2:**
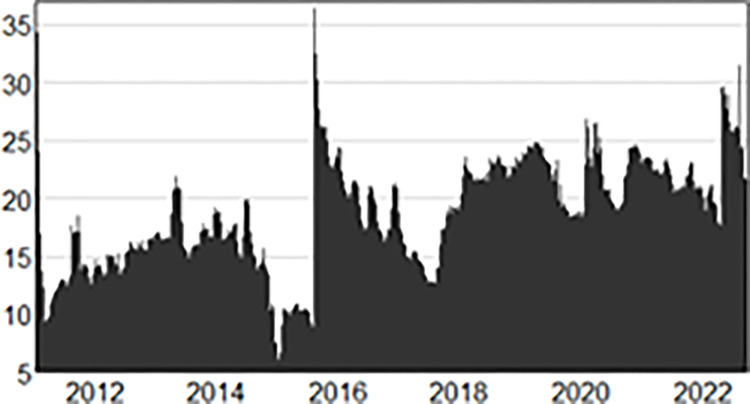
Total spillover index of international crude oil prices, onshore RMB exchange rate and offshore RMB exchange rate (%).

The content in Table 2 has presented the current situation of the three variables well. However, the deficiency of mean value conclusion is that it cannot effectively reflect the impact and influence of external events on variables, as well as the specific conditions of each variable in different stages. Therefore, the dynamic spillover results generated by the model is analyzed further. From Fig 2, during the entire sample period from 2011 to 2022, the international crude oil prices, onshore RMB exchange rate and offshore RMB exchange rate showed obvious time-varying characteristics, and four stages of small spillover peaks appeared successively after 2015, 2018, 2020 and 2022. For this changing trend, combined with the actual economic situation, the deep reasons can be stated from two aspects.

Firstly, the implementation of market-oriented reform of domestic exchange rate is conductive to improve the total spillover level. The market-oriented reform of RMB exchange rate started in 1994. With the *Decision of the CPC Central Committee on Several Issues Concerning the Establishment of a Socialist Market Economic System*, a single, managed floating exchange rate system based on market supply and demand was realized to set the main tone for the development of the exchange rate formation mechanism, which became an important milestone in the market-oriented reform of the exchange rate. In order to adapt to the economic development situation and actual demand better, the People’s Bank of China began to implement a managed exchange rate system with “in reference to a basket of currencies” as the core in July 2005. In August 2015, the People’s Bank of China further strengthened the role of market pricing mechanism, laying more emphasis on and emphasizing the development trend of the two-way fluctuation of RMB exchange rate and its ability to depreciate or rise. In Fig 2, the total spillover level of the three variables in August 2015 reached the maximum value of 37% during the sample period. In fact, the policy goal of the "8·11" exchange rate reform implemented by the People’s Bank of China is to expand the influence of the Renminbi, further increase the degree of participation of the Renminbi in the international market, and enhance its ability to reflect market conditions in the future. The results of Fig 2 are consistent with the research viewpoint of Li Zheng [24], the "8·11" exchange rate reform has significantly improved the level of Renminbi internationalization, and have led to an increase in the overall spillover index.

Secondly, international economic and financial events will also increase the total spillover level. In Fig 2, in addition to a peak of the total spillover index in 2015, there are also high spillover levels in 2018, 2020 and 2022. During this period, spillover levels reached 23%, 27% and 32%, respectively. The reviewing of the major international events in 2018, 2020 and 2022 shows that the main ones are the US-China trade dispute, COVID-19 pandemic, Ukraine crisis, and the interest rate hike of Federal Reserve. In August 2017, the United States announced an investigation against “China’s unfair trade practices”, that is, the so-called “301” investigation. In March 2018, the Office of the United States Trade Representative began imposing tariffs on Chinese goods worth of $60 billion after releasing its investigation results, marking the beginning of the trade dispute between China and the United States. In December 2019, the outbreak of the novel coronavirus epidemic impacted almost all countries in the world. Affected by the trade imbalances, export restrictions and the emergence of “deglobalization”, the global economy had a cliff-like decrease, and the international financial market was greatly impacted. The international environment in 2022 has become more complex, and the Ukraine crisis and the interest rates raising of the US Federal Reserve have a direct impact on the crude oil market and the exchange rates of major countries. The crude oil based international energy prices increased sharply and non-US currencies had substantial devaluation, which had once again exacerbated the chaos in the international financial market, and the total spillover fluctuation had significantly increased. In 2018, 2020 and 2022, the fluctuation in 2022 was significantly higher than that in 2018 and 2020. By combing through major international events during the sample period, it is found that crises caused by factors such as economy, trade, and geopolitics will exacerbate the volatility of international crude oil prices, onshore Renminbi exchange rate, and offshore Renminbi exchange rate.

### 3. Analysis of dynamic directional spillover effect

The trend of the total spillover index represents the overall characteristics of all variables. In order to better understand and study the influential effect of a single variable on all other variables and a single variable influenced by all other variables, it is also necessary to investigate and analyze the spillover level (To) and absorption level (From) of a single variable.

**Fig 3:**
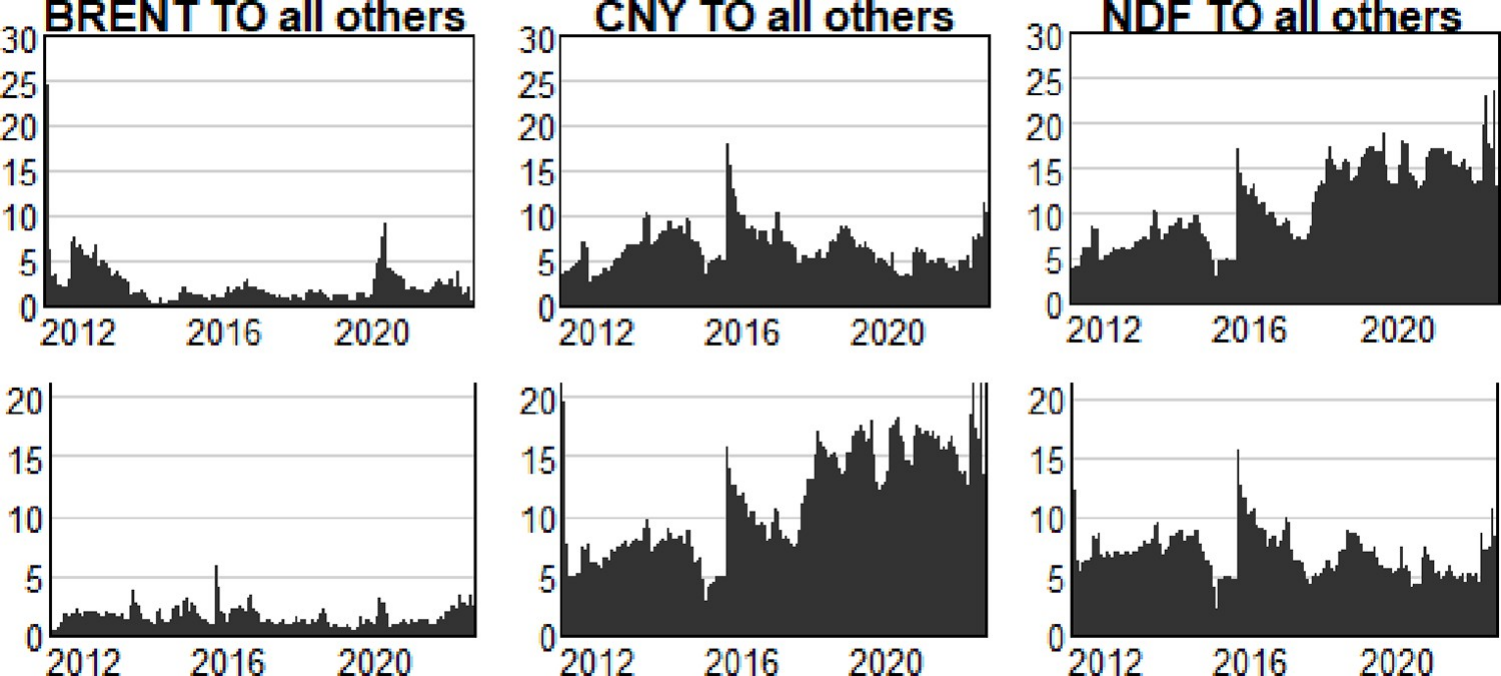
Directional spillover index of international crude oil prices, onshore RMB exchange rate and offshore RMB exchange rate (%).

Fig 3 provides more detailed variable spillover results. Consistent with the general situation in Fig 2, all variables in Fig 3 also show a trend of increasing spillover levels in 2015, 2018, 2020 and 2022. However, there are obvious differences of each variable in the performance. From the perspective of international crude oil prices, the level of spillover and absorption is not significant, basically around the level of 2%-3%, which means that there is a certain impact between crude oil prices and RMB exchange rate, but the impact is very limited. This paper believes that the factors leading to this limited impact come from two aspects: 1) The marketization reform of the Renminbi exchange rate is still in progress, and it is temporarily unable to fully reflect external shocks and influences, thus the transmission of oil prices is incomplete; 2) International crude oil is not only an important raw material base for countries around the world, but it also has been endowed with financial and political attributes. This leads to complex and variable factors influencing crude oil prices, such as investment behavior, supply scale, pricing currency, and strategic reserves, all of which will have an impact [25]. As a result, the Renminbi exchange rate’s impact on international crude oil prices is not significant, which is basically consistent with the views of Wu Lihua and Fu Chun [20] in their research. Compared with crude oil prices, the two-way fluctuation range of RMB exchange rate is more significant. Among them, the spillover level of offshore RMB exchange rate is significantly higher than that of the onshore RMB exchange rate, reaching the maximum in 2022, about 24%. The maximum spillover level of onshore RMB exchange rate is 17%, and it only occurred during the “exchange rate reform on August 11” in 2015. The core of the “8·11 Exchange Rate Reform” is the establishment of a pricing mechanism based on the “basket of currencies + closing rate”, enabling the RMB exchange rate to have greater participation in international financial markets. Compared to the earlier implementation of exchange rate reform policies, the “8·11” RMB has shown a significant increase in spillover effects [26]. From the current situation, it appears that the offshore RMB exchange rate is more susceptible to the influence of the international market compared to the onshore RMB exchange rate.

### 4. Dynamic net spillover effect analysis

**Fig 4:**
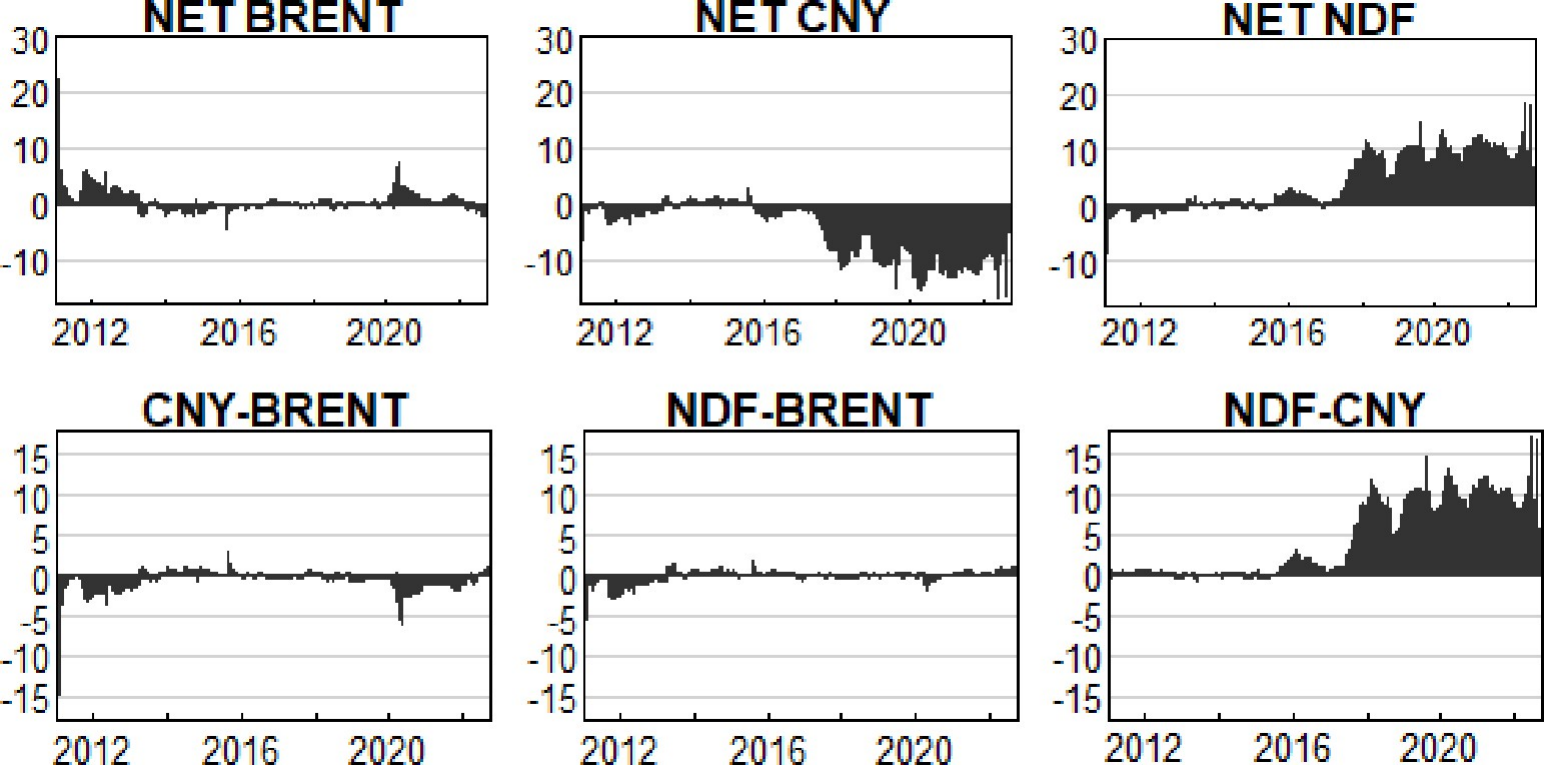
Net spillover index of international crude oil prices, onshore RMB exchange rate and offshore RMB exchange rate (%).

TVP-VAR-DY model also provides the results of net spillover level of each variable itself, and its established core is the net value obtained from the variable spillover level excluding the absorption level. In Fig 4, Net spillover level (Net) of the three variables and the mutual net spillover level between the two variables are presented respectively.

By combining the trends of international crude oil prices, onshore RMB exchange rate and offshore RMB exchange rate in Figs 3 and 4, the net spillover index in Fig 4 reveals three more detailed conclusions: 1)Although the “exchange rate reform on August 11” in 2015 has improved the marketization level of onshore RMB exchange rate, which is conducive to improving the influence of RMB exchange rate in the future and its long-term development, it also means that onshore RMB exchange rate may face more external shocks and impacts to a certain extent. In most time after the “exchange rate reform on August 11”, onshore RMB exchange rate has shown obvious negative net spillover effect, which has increased year by year since 2015 and reached a maximum of -18% in 2022. The conclusion of this study is consistent with the views of Li Zheng et al. [27] and Zhang Yingying [28]. Compared to the offshore RMB exchange rate, the onshore RMB exchange rate plays a more predominant role as a recipient in the international market. 2) Both onshore RMB exchange rate and offshore RMB exchange rate have a very limited impact on the international crude oil prices, but they are inconsistent in their impact results. However, the year 2020 is a more important turning point. Before 2020, the performance of the two is similar, that is, the period from 2011 to 2014 is mainly reflected as small negative, and the period from 2014 to 2020 is reflected as small positive. Since 2020, onshore RMB exchange rate has mainly shown negative effect, while offshore RMB exchange rate has shown positive effect. This means that the current impact of RMB exchange rate on crude oil is mainly achieved through the offshore market. 3)The spillover of onshore RMB exchange rate is largely driven by offshore RMB exchange rate, which is consistent with the research conclusions of Zhang Yingying [29], Wang Zhiyong and Guo Jingbo [30]. The above scholars show in their research that the current offshore RMB exchange rate has stronger ability to transmit information than onshore RMB exchange rate.

### 5. Robustness test

To ensure the robustness of the research conclusions, the model was tested by adding variables in this paper. According to Beckmann et al. [31], stock price, as an exogenous variable, will also play a certain role in the transmission process of international crude oil prices, onshore RMB exchange rate and offshore RMB exchange rate. Therefore, this paper includes the MSCI China A-share index from January 4, 2011 to September 30, 2022 into the research framework by establishing A TVP-VAR-DY model including four variables. According to the selection criteria of the optimal lag order, nlag = 2 and nfore = 10 are substituted into the model. The results are generally consistent with those above (see S1 Appendix).

## VI. Conclusions and policy recommendations

TVP-VAR-DY model is used in this paper to study the fluctuation spillover effects between international crude oil prices, onshore RMB exchange rate and offshore RMB exchange rate from 2011 to 2022. Through empirical test, the following conclusions are made: Overall, there is a significant time-varying relationship between international crude oil prices, onshore RMB exchange rate and offshore RMB exchange rate, and this total spillover relationship will be enhanced under the influence of domestic policy reform and international economic and financial emergencies. From the specific performance of each variable, the spillover and absorption levels between international crude oil prices, onshore exchange rate and offshore RMB exchange rate are not particularly significant. The exchange rate results show that the net spillover level of offshore RMB exchange rate is significantly higher than that of onshore RMB exchange rate, and RMB exchange rate spillover is mainly driven by offshore RMB exchange rate to a large extent.

Based on the above research conclusions, the following policy recommendations are put forward in this paper.

### 1. Advance the development and utilization of new energy in an orderly manner, and reduce the adverse impact of oil price fluctuation

With more complex international economic situation, the trend of large fluctuation in international crude oil prices still exists due to uncertainty under the imbalance between supply and demand. In consideration of China’s share in the crude oil reserve and consumption market, it is necessary to proactively invest in clean energy and renewable energy, fully harness the advantages of wind energy in desert and barren areas, utilize regions abundant in solar energy for photovoltaic power generation, promote comprehensive utilization of nuclear energy, and prepare for pumped storage power stations. These measures aim to achieve high-quality and sustainable energy utilization. Alongside the process of energy transformation and upgrading, efforts should be made to shift the role of crude oil from primary energy to a more adjustable energy source.

### 2. Actively promote market-oriented reform of RMB exchange rate and give play to the guiding role of offshore market

In order to ensure economic stability, it is important to gradually increase the fluctuation range of the RMB exchange rate in an orderly manner, forming an awareness and expectation of two-way fluctuations, and enabling the RMB exchange rate to serve as a shock absorber for absorbing internal and external impacts. At the same time, it is necessary to fully utilize the international platforms provided by Shanghai and Hong Kong as international financial centers, offering more exchange channels and meeting the demands of domestic and foreign enterprises and individuals, thus achieving synchronized development of onshore and offshore markets. It should be noted that with the increased level of RMB marketization, the effects of cross-border capital flows, two-way capital movement, and external risk shocks may be magnified. Therefore, it is important to conduct early risk monitoring and management.

### 3. Accelerate the construction of the crude oil-RMB settlement system

The establishment of a crude oil-RMB settlement payment system is fundamental to mitigating the impact of international crude oil price fluctuations. Actively promoting the regionalization of the RMB and implementing RMB settlements in trade activities along the Belt and Road countries, ASEAN, and BRICS nations will lay the foundation for the construction of the crude oil-RMB system. Additionally, maintaining good diplomatic relations with major oil-producing countries such as Iran, the United Arab Emirates, and Russia, will help drive the scale of crude oil-RMB settlements and establish a closed-loop circulation system for crude oil-RMB.

Overall, there is a certain degree of linkage between international crude oil prices and the RMB exchange rate. This research conclusion not only provides insights into the potential impact of international crude oil price fluctuations on China’s economy but also explains the mechanisms through which the two exchange rates reflect each other. However, the financial market is a complex and dynamic environment, and with the acceleration of China’s financial opening, future research needs to consider more factors such as stock prices, interest rates, and international balance of payments and their role in this linkage.

## Supporting information

S1 Appendix Robustness test results. (DOCX)

S1 Data (XLSX)
