# Supplementary material for: Estimating the effect of international crude oil prices on RMB exchange rate with two-way fluctuation spillover
Source: PLoS One. 2023 Oct 10;18(10):e0292615. doi: 10.1371/journal.pone.0292615 (PMC10564126; doi:10.1371/journal.pone.0292615)
Supplement: S1 Appendix — (DOCX) [file pone.0292615.s001.docx]

**Appendix: Robustness test results**

**Table 1. Static spillover effect of international crude oil prices, onshore RMB exchange rate, offshore RMB Exchange rate and stock price (%)**

|  | **BRENT** | **CNY** | **NDF** | **MSCI** | **FROM** | **TCI** |
| --- | --- | --- | --- | --- | --- | --- |
| **BRENT** | 92.9 | 1.3 | 2.4 | 3.4 | 7.1 |  |
| **CNY** | 2.8 | 65.4 | 27.7 | 4.2 | 34.6 |  |
| **NDF** | 2.6 | 15.7 | 76.8 | 4.9 | 23.2 |  |
| **MSCI** | 3.1 | 1.9 | 5.5 | 89.5 | 10.5 |  |
| **TO** | 8.4 | 18.9 | 35.6 | 12.4 | 75.4 |  |
| **NET** | 1.3 | -15.7 | 12.4 | 1.9 |  |  |
| **TCI** |  |  |  |  |  | 18.9 |


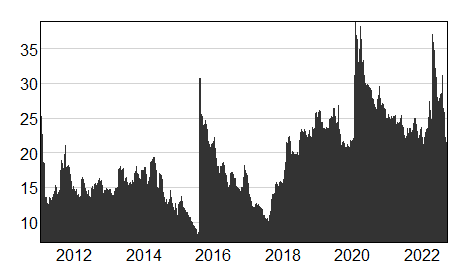


**Figure 1. Total spillover index of international crude oil prices, onshore RMB exchange rate, offshore RMB exchange rate and stock price (%)**


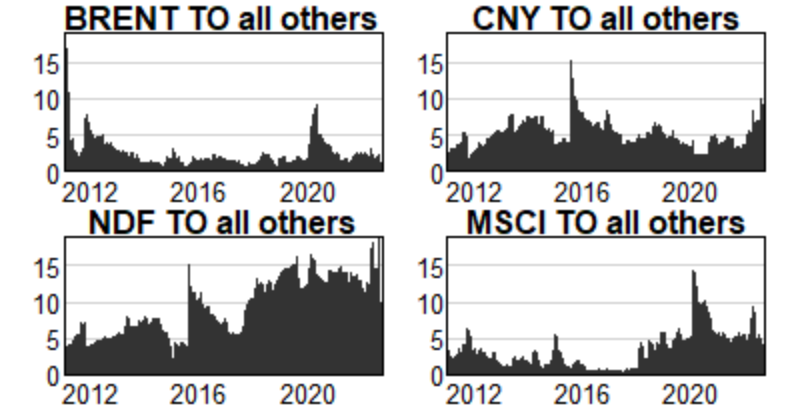


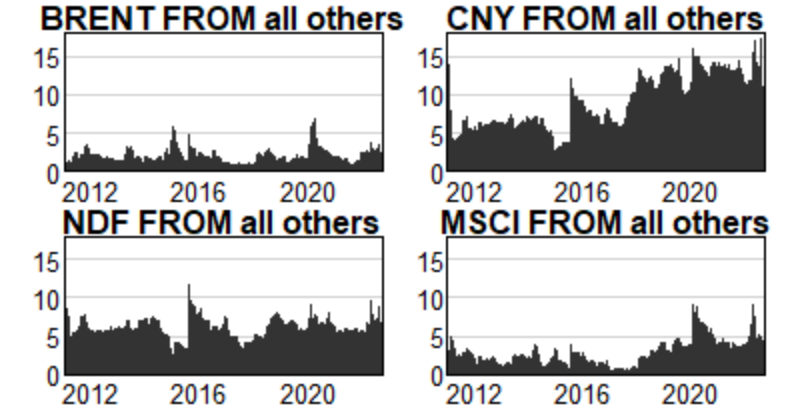


**Figure 2. Directional spillover index of international crude oil prices, onshore RMB exchange rate, offshore RMB exchange rate and stock price (%)**


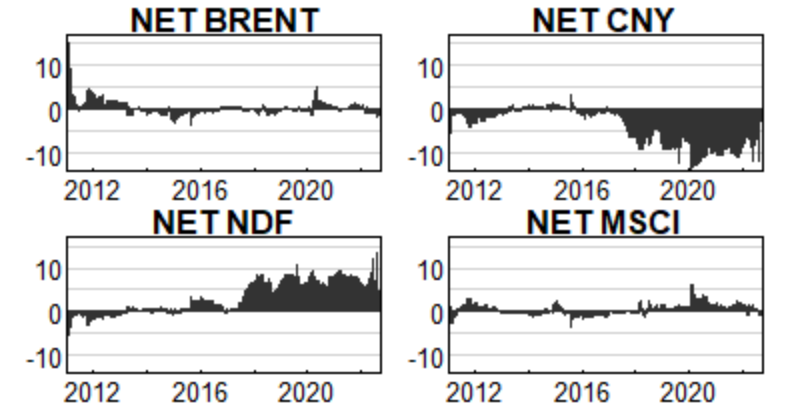


**Figure 3. Net spillover index of international crude oil prices, onshore RMB exchange rate, offshore RMB exchange rate and stock price (%)**


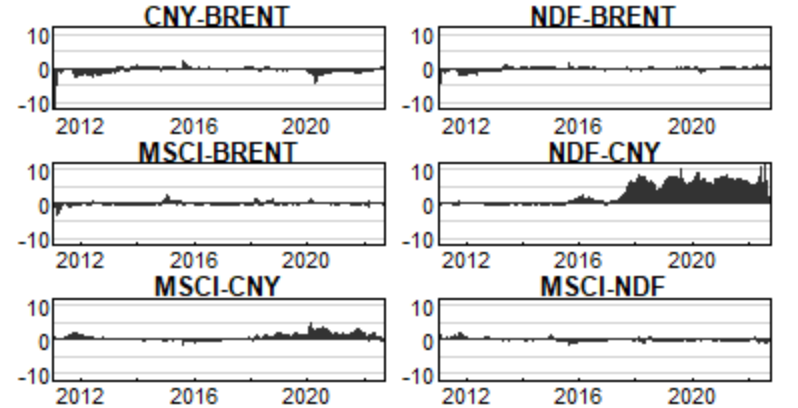


**Figure 4. Mutual net spillover index of international crude oil prices, onshore RMB exchange rate, offshore RMB exchange rate and stock price (%)**
